# Supplementary material for: sincFold: end-to-end learning of short- and long-range interactions in RNA secondary structure
Source: Brief Bioinform. 2024 Jun 10;25(4):bbae271. doi: 10.1093/bib/bbae271 (PMC11163250; doi:10.1093/bib/bbae271)

sincFold: end-to-end learning of short- and long-range interactions in RNA secondary structure

Supplementary Material

1 Hyperparameter search

The exploration of hyperparameters on the Ablation dataset resulted in a set of 4021 runs with different parameters and an average F_1 score. A random forest regressor was trained on this data to explore the hyperparameter effect (predictability) on F_1. Using the mean decrease of impurity [1] (Figure S1) the hyperparameters batch size, learning rate (LR), use of learning rate schedule, weights λ_β, λ_{L_1} and the architecture of the ResNet stages were analyzed and selected iteratively. The LR, ResNet2D kernel size, bottleneck filters on the first ResNet layer (Bottleneck$_1$), batch size and number of layers in the ResNet2D resulted in the most critical parameters, in the sense that poor choices may result in strong loss of performance (Figure S2). Optimal LR found was 0.001 and batch size less than 8. In the ResNet2D stage, 2 layers with kernel size 7 were the best choice. Finally, it was better to use a small kernel of size 3 in ResNet1D.

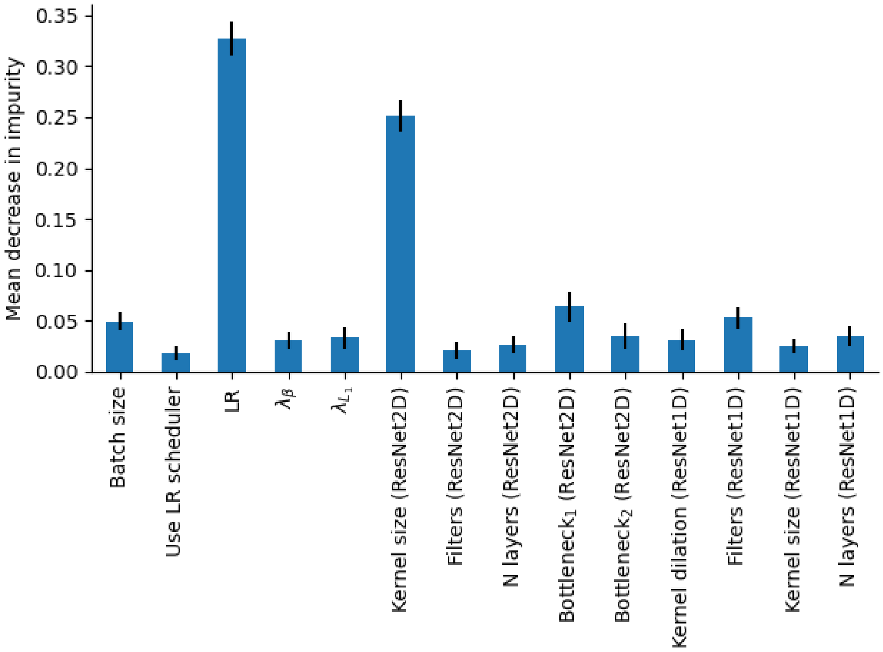

Figure 1: Hyperparameter importance based on the mean decrease in impurity of the random forest regressor. Higher values indicate more predictability (correlation) between hyperparameter change and resulting F_1 score.

[1] Ishwaran, H. (2015). The effect of splitting on random forests. Mach Learn., 99(1), 75–118.

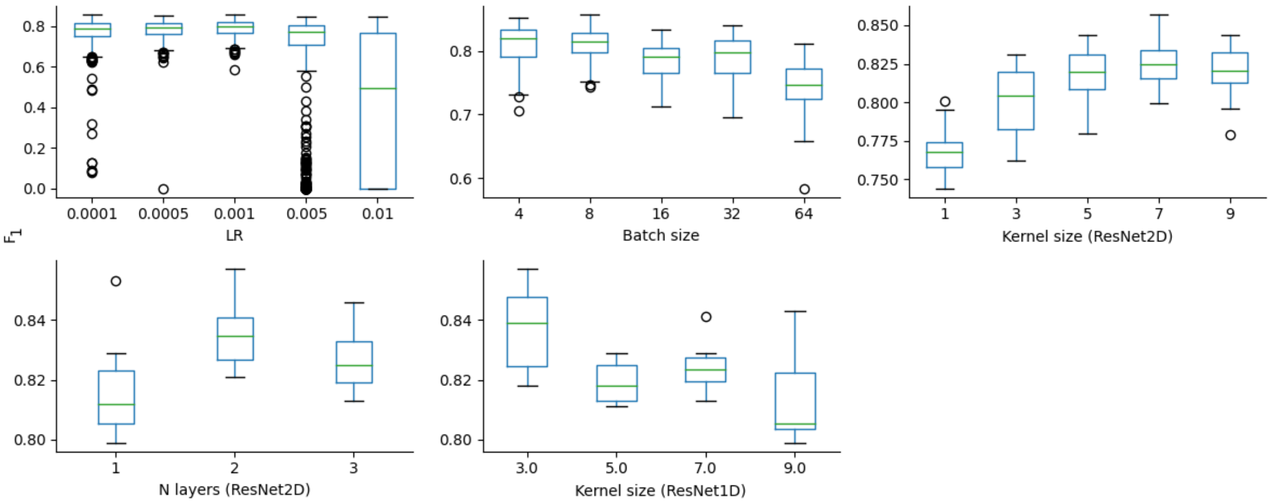

Figure 2: Impact on F_1 score of the critical hyperparameters of sincFold model.

2 Inter-family results

Table 1: All metrics for the inter-family experiment.

			grp1	tmRNA	tRNA	5s	srp	telom.	RNaseP	16s	23s
family size			74	462	557	1283	914	35	454	65	15
ave(L)			375	366	77	119	180	438	332	317	326
ave(min(structural distance))			0.526	0.508	0.445	0.497	0.548	0.531	0.519	0.490	0.574
F_1											
	Classical methods	RNAfold	0.540	0.419	0.677	0.614	0.593	0.465	0.527	0.532	0.694
		RNAstructure	0.494	0.409	0.690	0.584	0.568	0.443	0.532	0.533	0.663
		IPknot	0.587	0.394	0.705	0.692	0.588	0.517	0.556	0.580	0.689
		LinearPartition-C	0.592	0.370	0.699	0.718	0.589	0.516	0.560	0.594	0.718
		LinearPartition-V	0.545	0.404	0.658	0.632	0.596	0.453	0.554	0.550	0.717
		LinearFold-C	0.545	0.390	0.673	0.726	0.589	0.490	0.497	0.565	0.657
		LinearFold-V	0.545	0.390	0.673	0.726	0.589	0.490	0.497	0.565	0.657
		ProbKnot	0.535	0.415	0.680	0.599	0.573	0.465	0.572	0.558	0.692
	Hybrid	MXfold2	0.459	0.411	0.531	0.555	0.561	0.363	0.482	0.520	0.594
	DL	UFold	0.429	0.347	0.492	0.377	0.199	0.177	0.421	0.332	0.394
		REDfold	0.311	0.274	0.415	0.472	0.179	0.112	0.358	0.366	0.402
		sincfold	0.350	0.350	0.685	0.439	0.250	0.154	0.443	0.392	0.402
INF											
	Classical methods	RNAfold	0.541	0.420	0.677	0.613	0.593	0.471	0.529	0.534	0.694
		RNAstructure	0.496	0.410	0.689	0.584	0.568	0.449	0.533	0.535	0.664
		IPknot	0.588	0.396	0.707	0.692	0.589	0.520	0.560	0.582	0.691
		LinearPartition-C	0.592	0.373	0.700	0.718	0.590	0.520	0.563	0.595	0.719
		LinearPartition-V	0.547	0.406	0.658	0.632	0.597	0.459	0.556	0.553	0.718
		LinearFold-C	0.546	0.393	0.678	0.728	0.590	0.494	0.500	0.567	0.659
		LinearFold-V	0.546	0.393	0.678	0.728	0.590	0.494	0.500	0.567	0.659
		ProbKnot	0.536	0.416	0.681	0.598	0.573	0.470	0.573	0.560	0.692
	Hybrid	MXfold2	0.459	0.412	0.530	0.554	0.561	0.365	0.483	0.521	0.593
	DL	UFold	0.434	0.350	0.515	0.408	0.209	0.177	0.438	0.345	0.415
		REDfold	0.317	0.278	0.442	0.498	0.189	0.114	0.374	0.377	0.421
		sincfold	0.362	0.356	0.696	0.475	0.260	0.160	0.449	0.397	0.430
WL											
	Classical methods	RNAfold	0.673	0.591	0.765	0.717	0.712	0.610	0.666	0.685	0.773
		RNAstructure	0.642	0.582	0.776	0.697	0.695	0.598	0.669	0.684	0.758
		IPknot	0.716	0.599	0.781	0.771	0.712	0.658	0.695	0.733	0.771
		LinearPartition-C	0.725	0.592	0.791	0.796	0.719	0.659	0.701	0.750	0.793
		LinearPartition-V	0.672	0.577	0.750	0.725	0.711	0.596	0.681	0.692	0.786
		LinearFold-C	0.707	0.606	0.783	0.809	0.724	0.644	0.668	0.739	0.763
		LinearFold-V	0.707	0.606	0.783	0.809	0.724	0.644	0.668	0.739	0.763
		ProbKnot	0.670	0.586	0.760	0.709	0.699	0.613	0.691	0.696	0.766
	Hybrid	MXfold2	0.632	0.599	0.673	0.690	0.692	0.562	0.644	0.688	0.720
	DL	UFold	0.644	0.579	0.675	0.628	0.517	0.528	0.636	0.616	0.623
		REDfold	0.595	0.566	0.658	0.669	0.518	0.541	0.612	0.646	0.628
		sincfold	0.610	0.582	0.783	0.653	0.538	0.579	0.631	0.627	0.631

3 Performance on pseudoknots and folding motifs

Table 2: Performance for methods on pseudoknots and folding motifs in bpRNA TS0

F_1	**RNAfold**	**LinearPartition-C**	**MXfold2**	**REDfold**	**Ufold**	**sincFold**
All base-pairs	0.508	0.547	0.502	0.621	0.580	**0.631**
Pseudoknots	0.424	0.466	0.435	0.532	0.489	**0.582**
Stems	0.658	0.670	0.594	0.699	**0.716**	0.688
Hairpin loops	0.490	0.356	0.456	0.490	0.278	**0.599**
Bulges	0.720	0.755	0.781	0.797	0.697	**0.861**
Internal loops	0.426	0.457	0.513	0.484	0.412	**0.634**
Dangling end	0.414	0.556	0.684	0.682	0.524	**0.764**
Multi-loops	0.654	0.694	0.731	0.781	0.707	**0.855**

4 RNA secondary structure predictions

The following pages show sample predictions of the methods in the inter-family cross-validation experiment. In the case of *Bacillus anthracis* ThiI, this is a sequence outside the ArchiveII dataset.

Chlamydomonas reinhardtii mito domain4

Reference

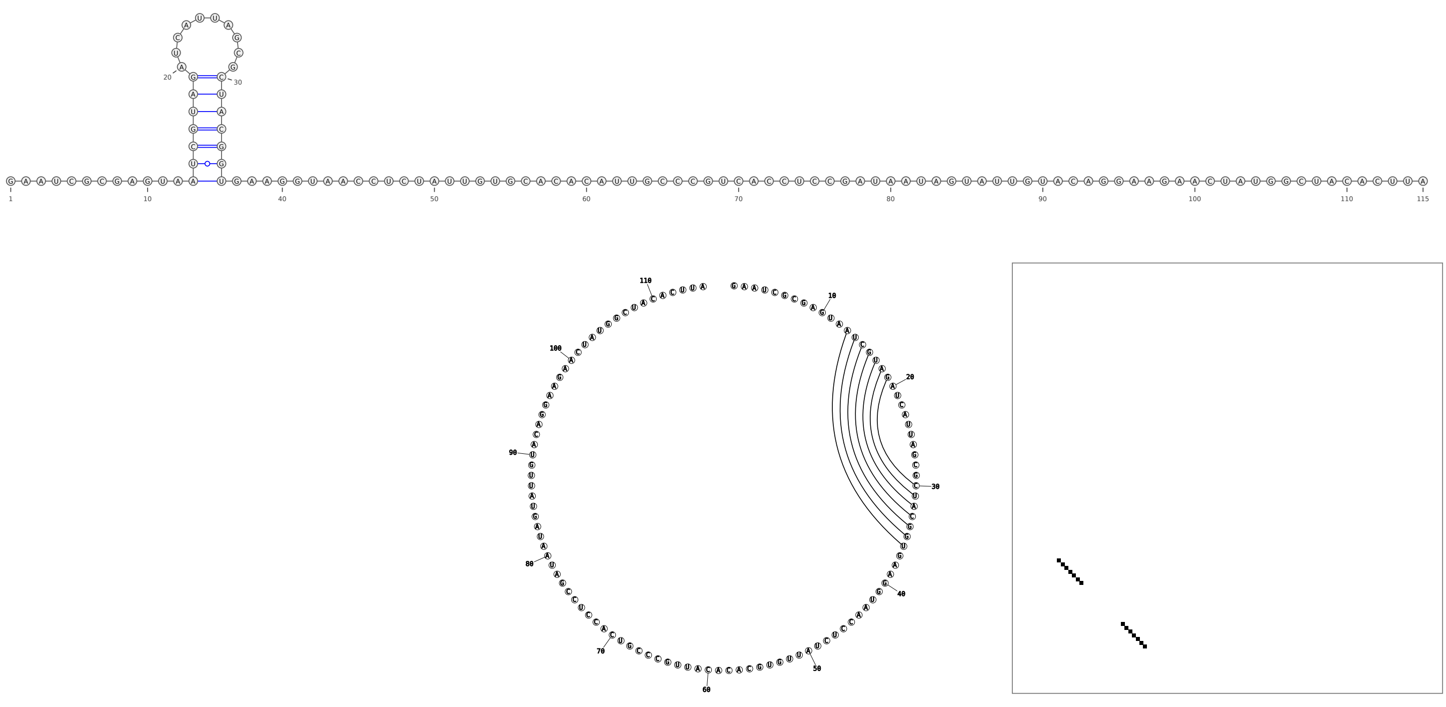

RNAfold $F_1 = 0.00$

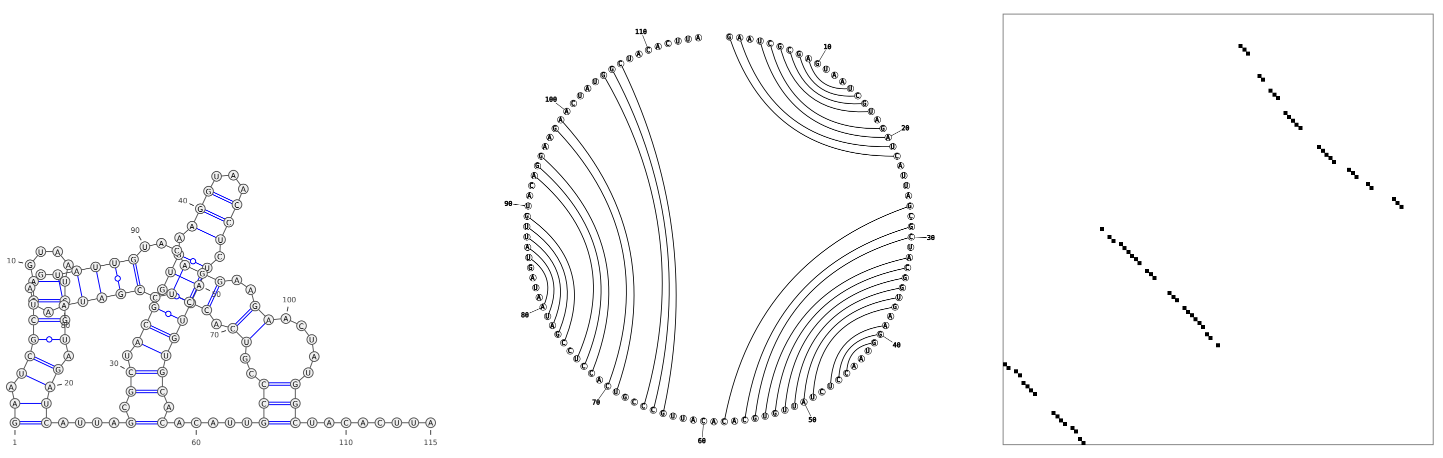

LinearPartition-C $F_1 = 0.00$

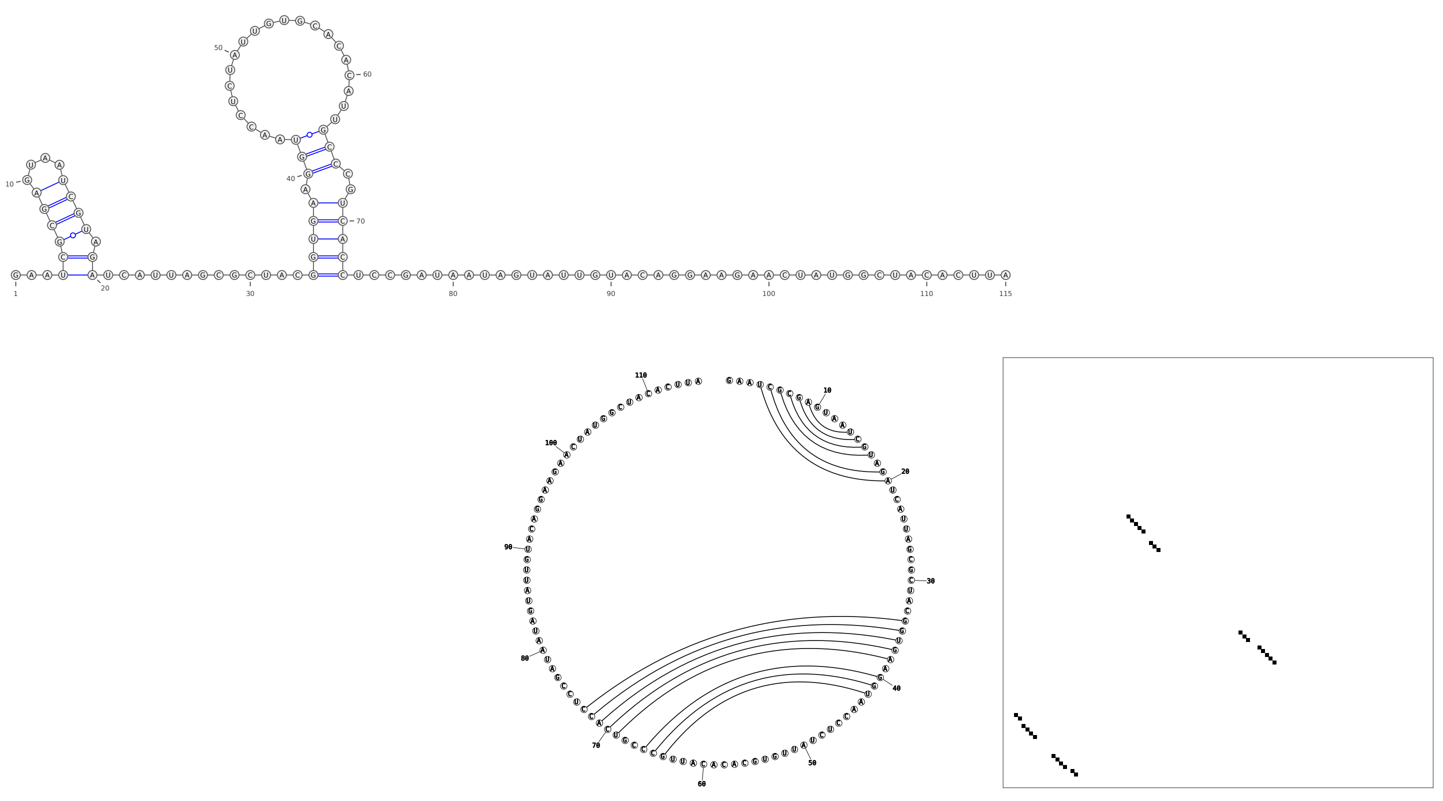

MXfold2 $F_1 = 0.26$

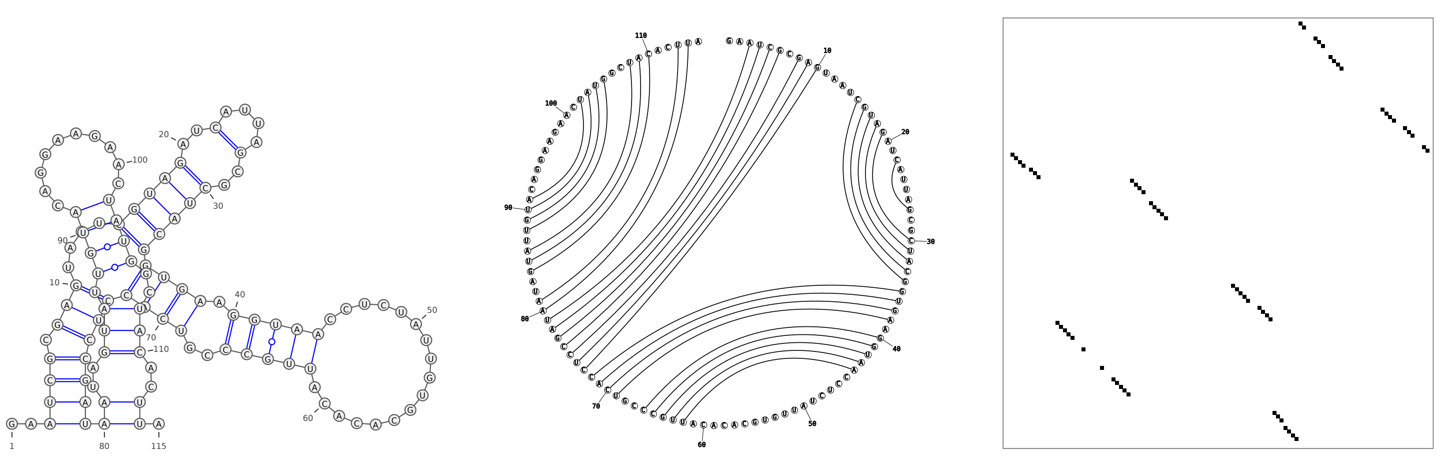

REDfold $F_1 = 0.44$

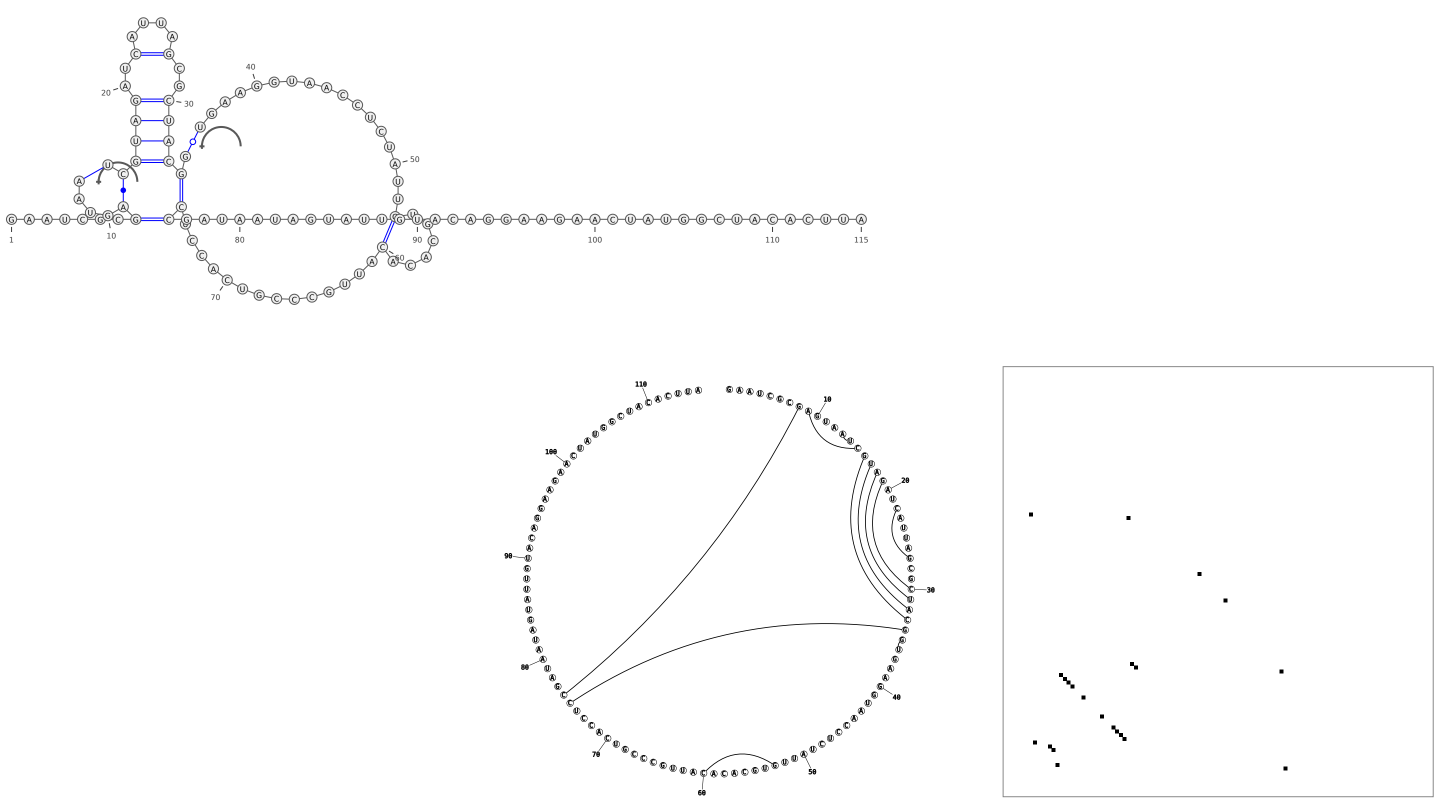

UFold $F_1 = 0.00$

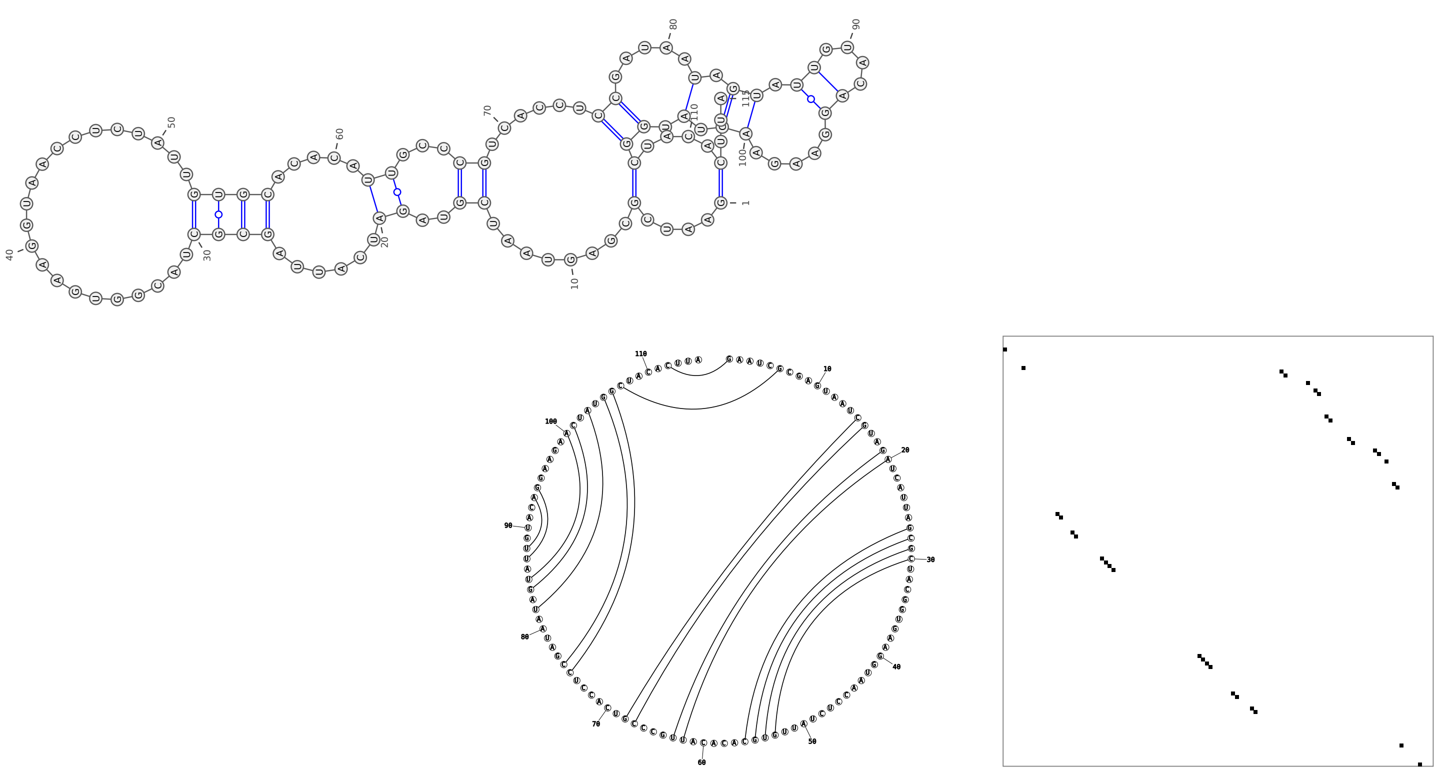

sincFold $F_1 = 0.73$

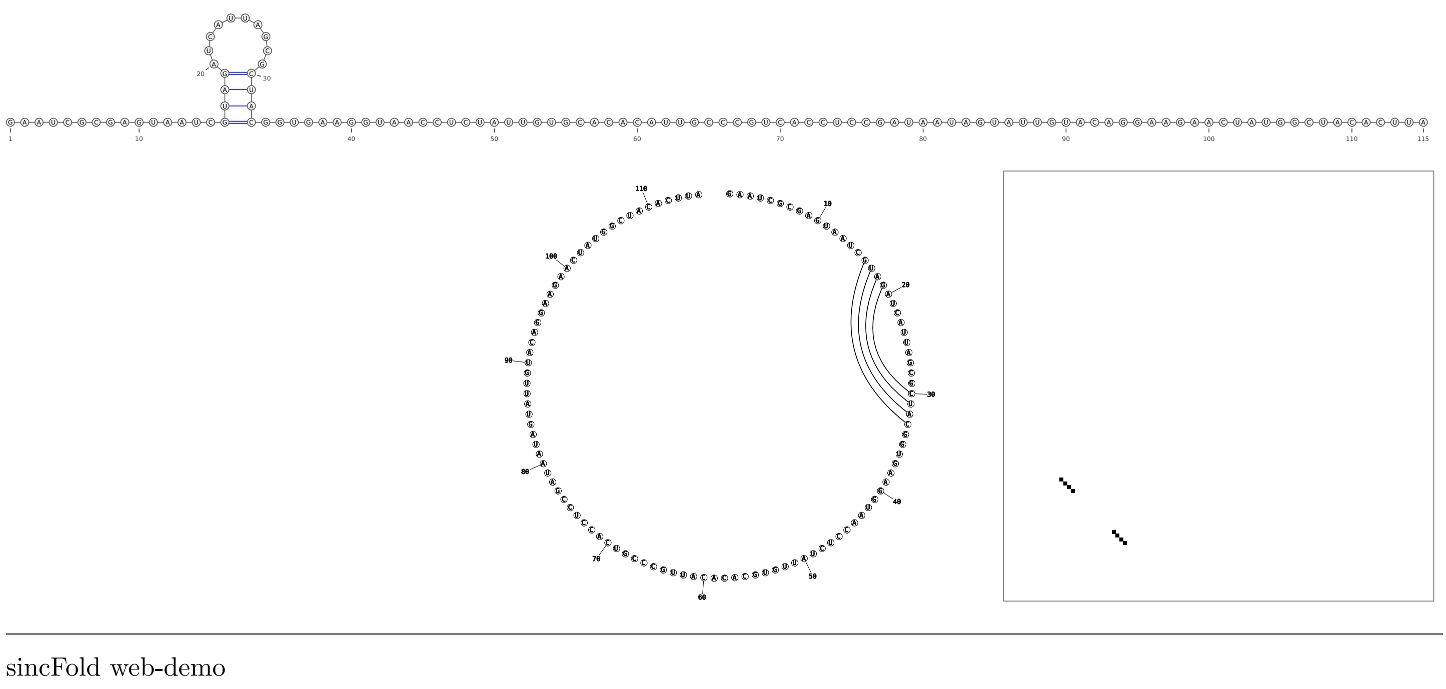

sincFold web-demo

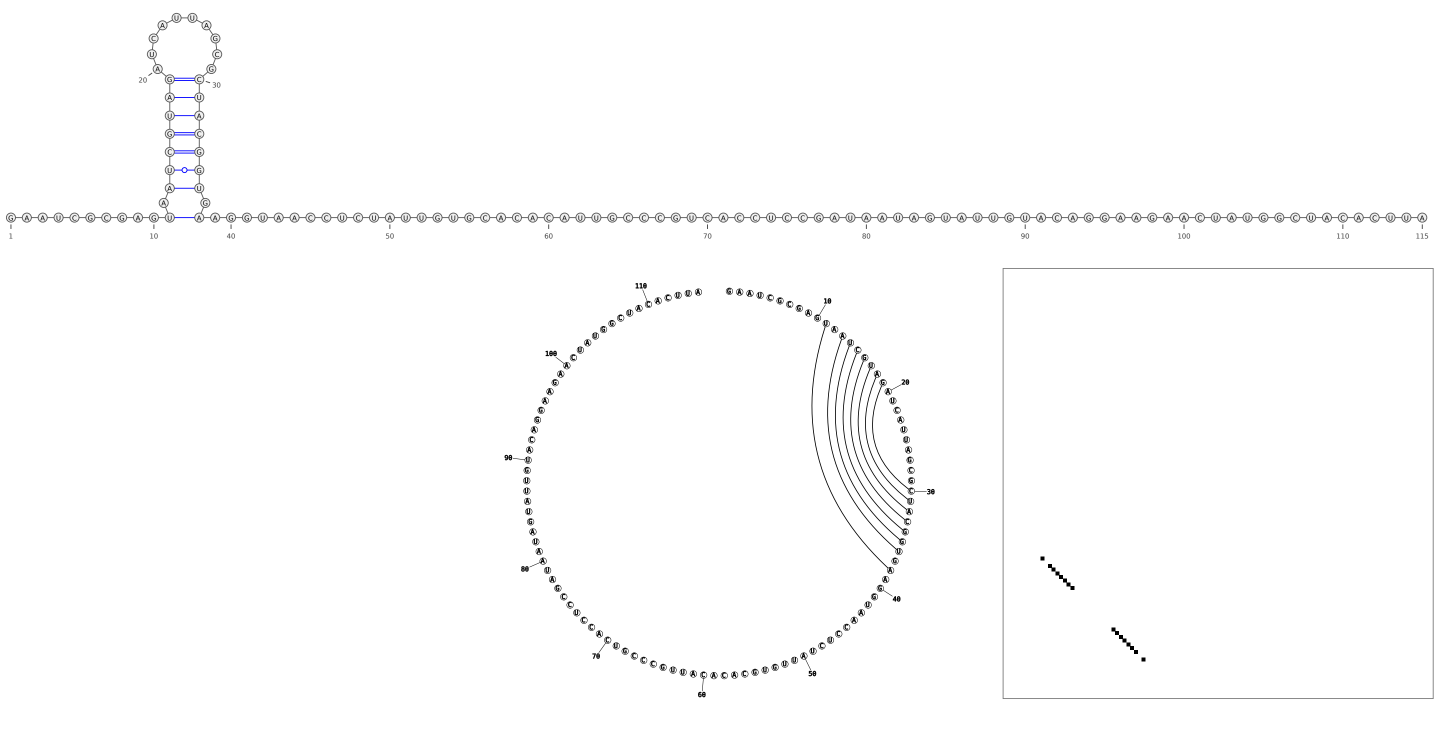

Bacillus anthracis ThiI

Reference

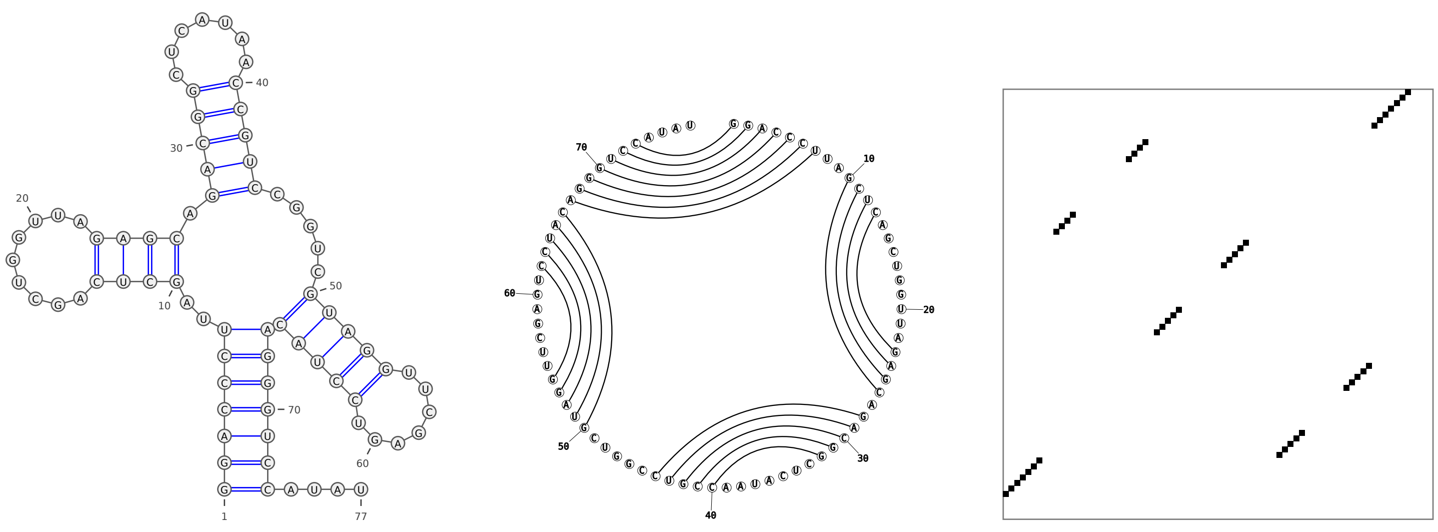

RNAfold $F_1 = 0.57$

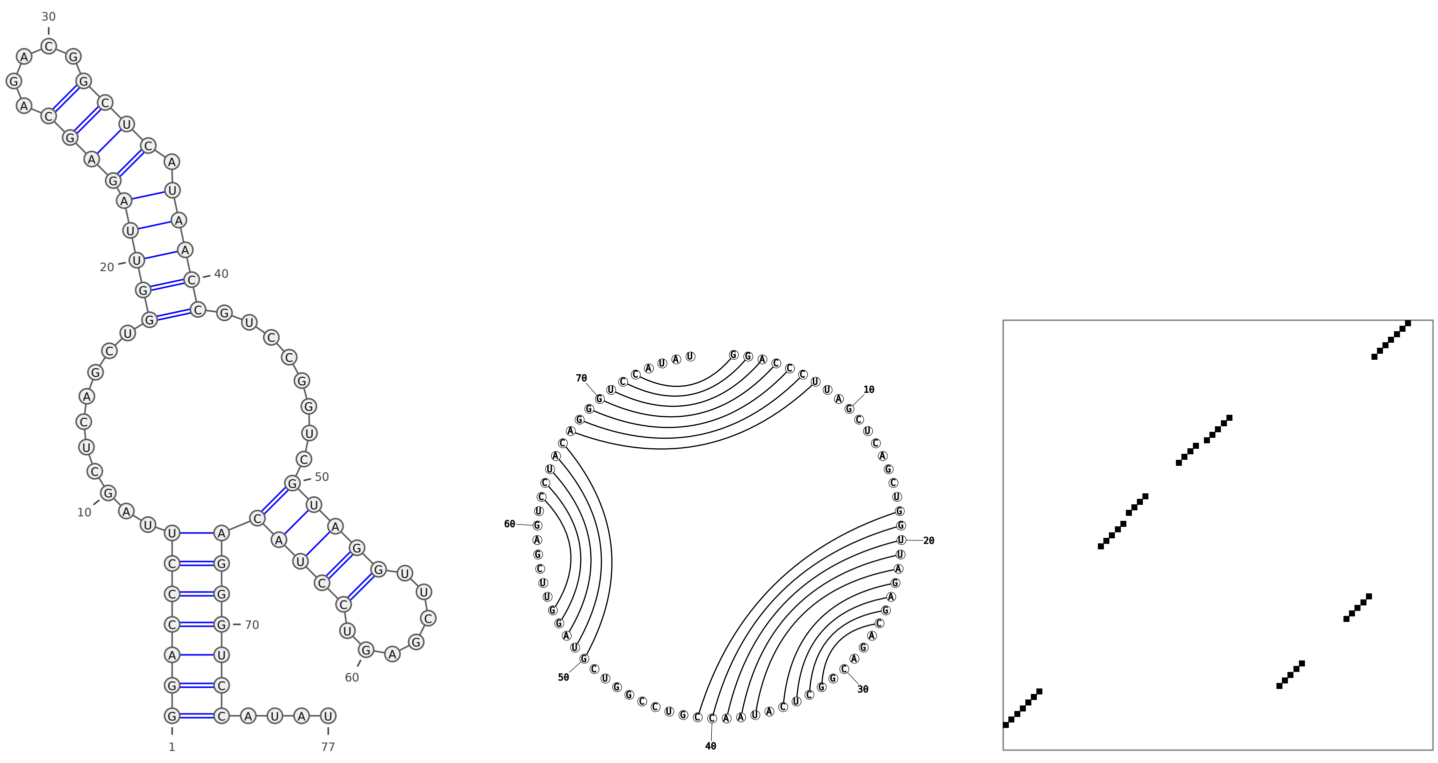

LinearPartition-C $F_1 = 0.51$

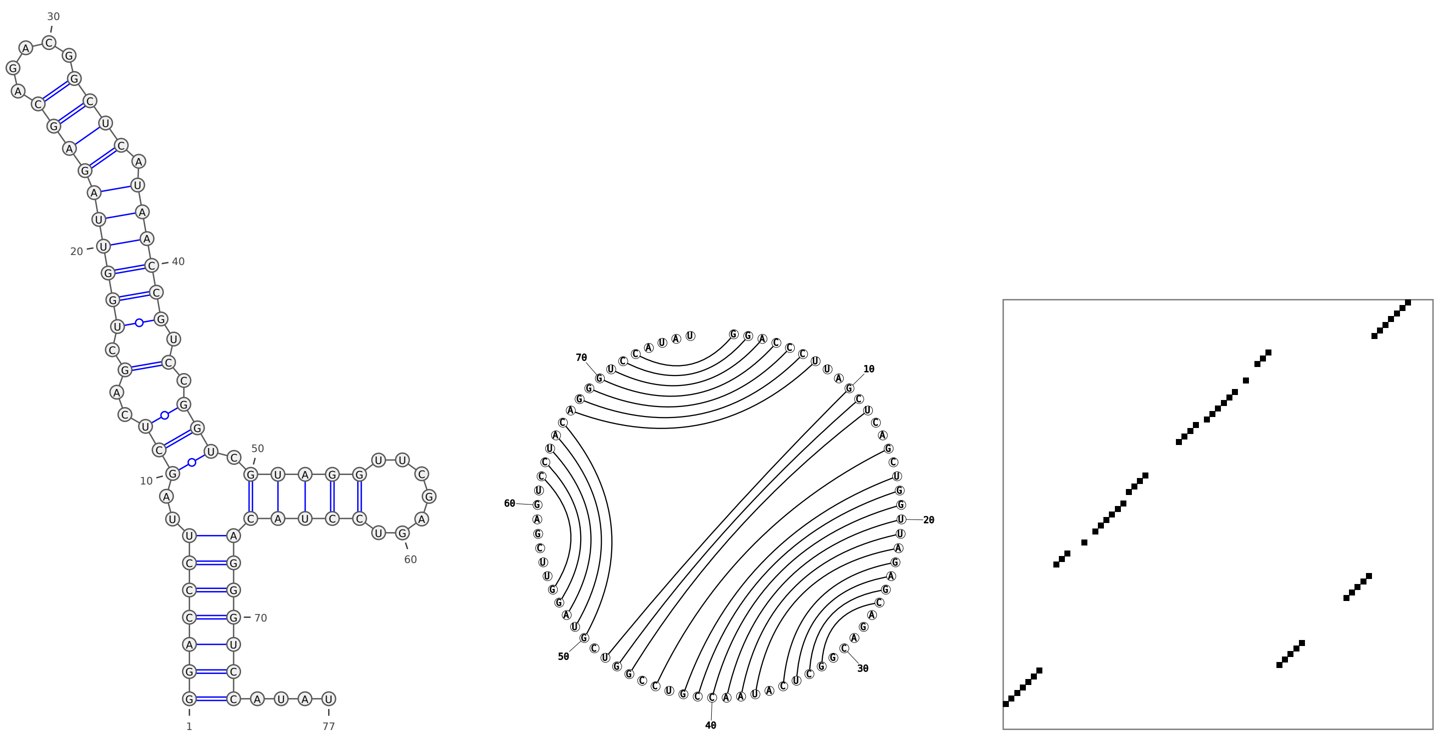

MXfold2 $F_1 = 0.55$

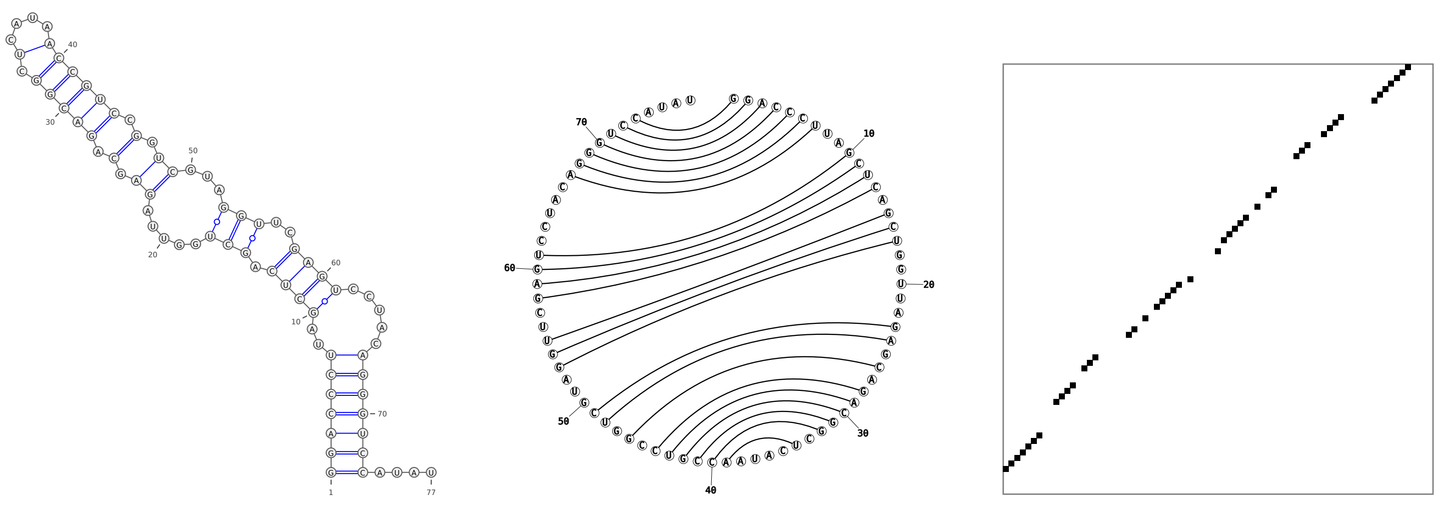

REDfold $F_1 = 0.67$

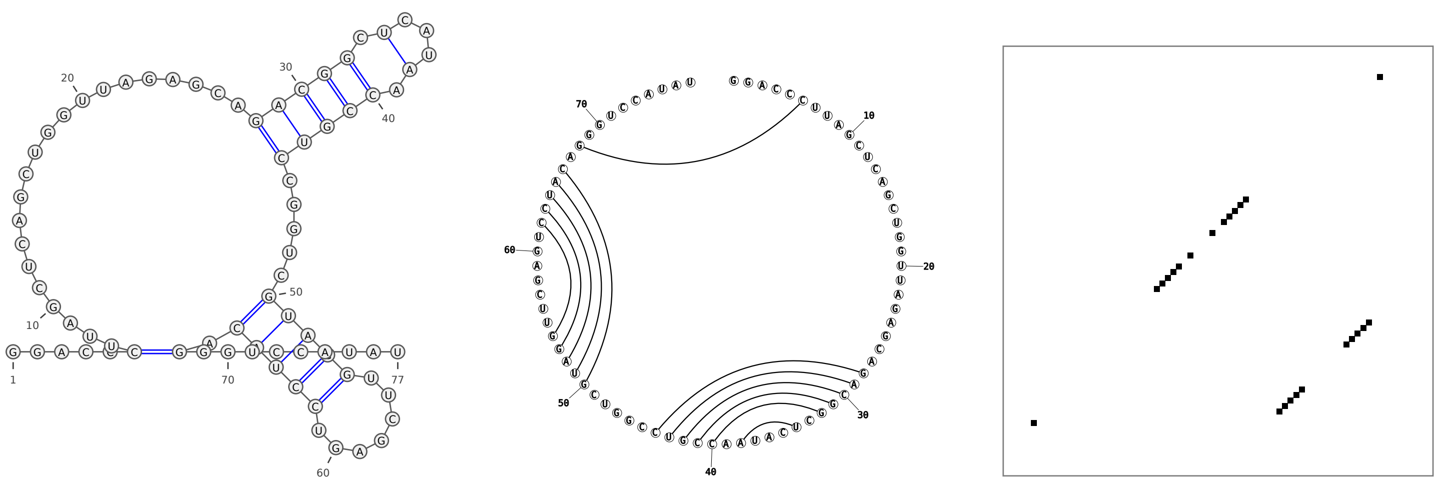

UFold $F_1 = 0.62$

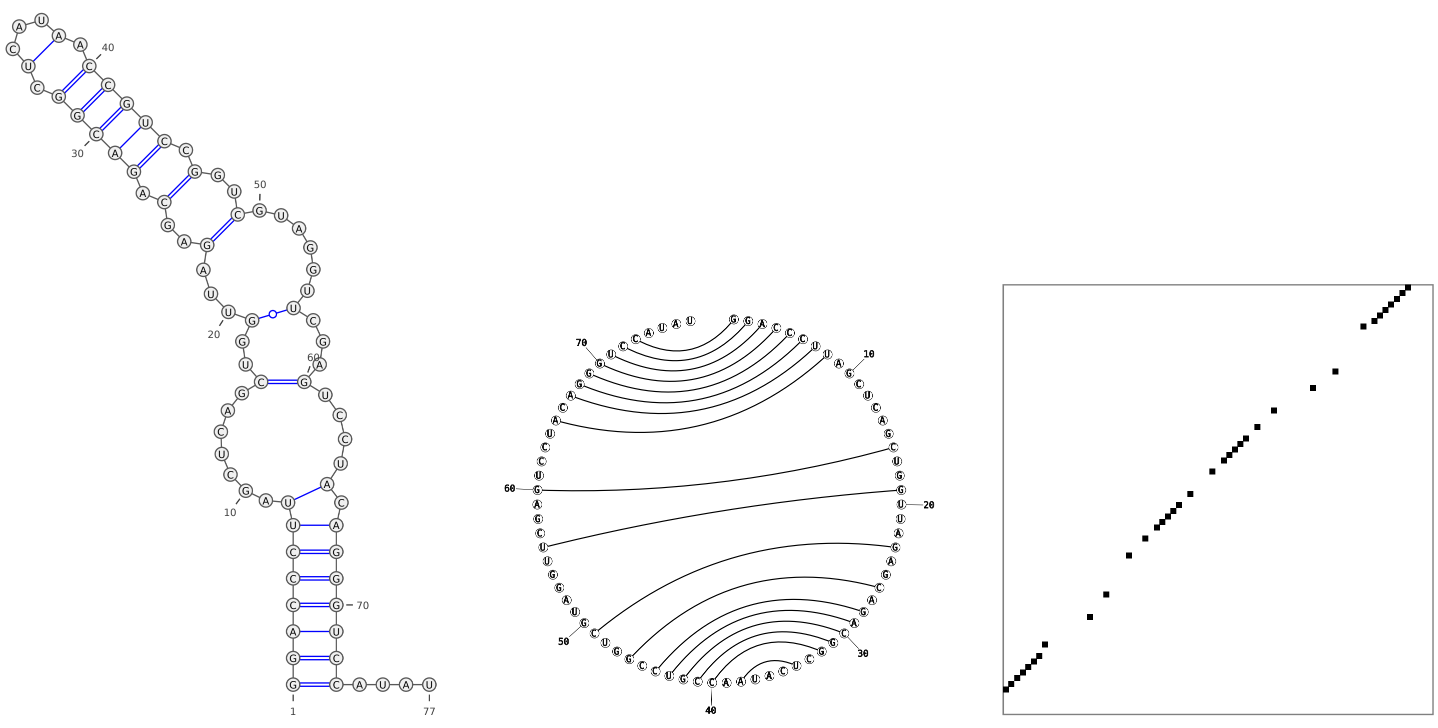

sincFold $F_1 = 0.74$

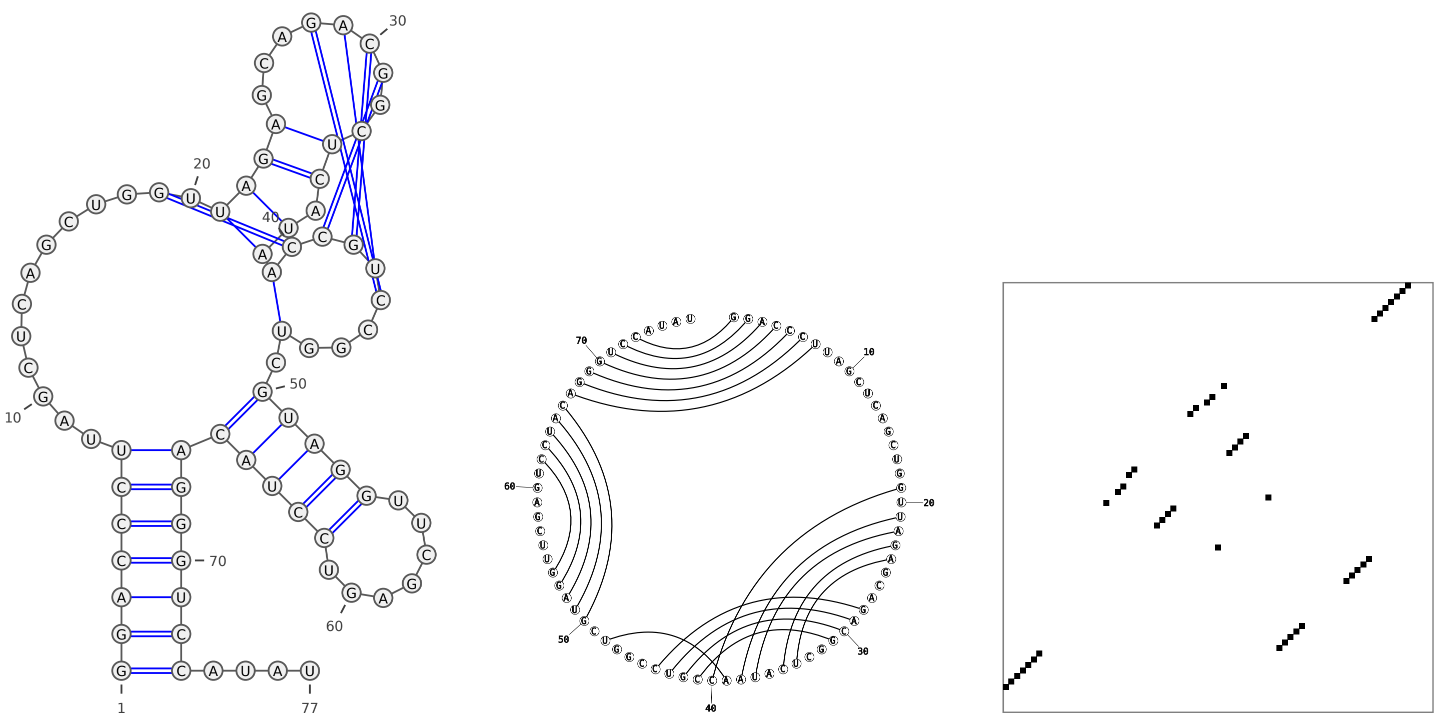

sincFold web-demo

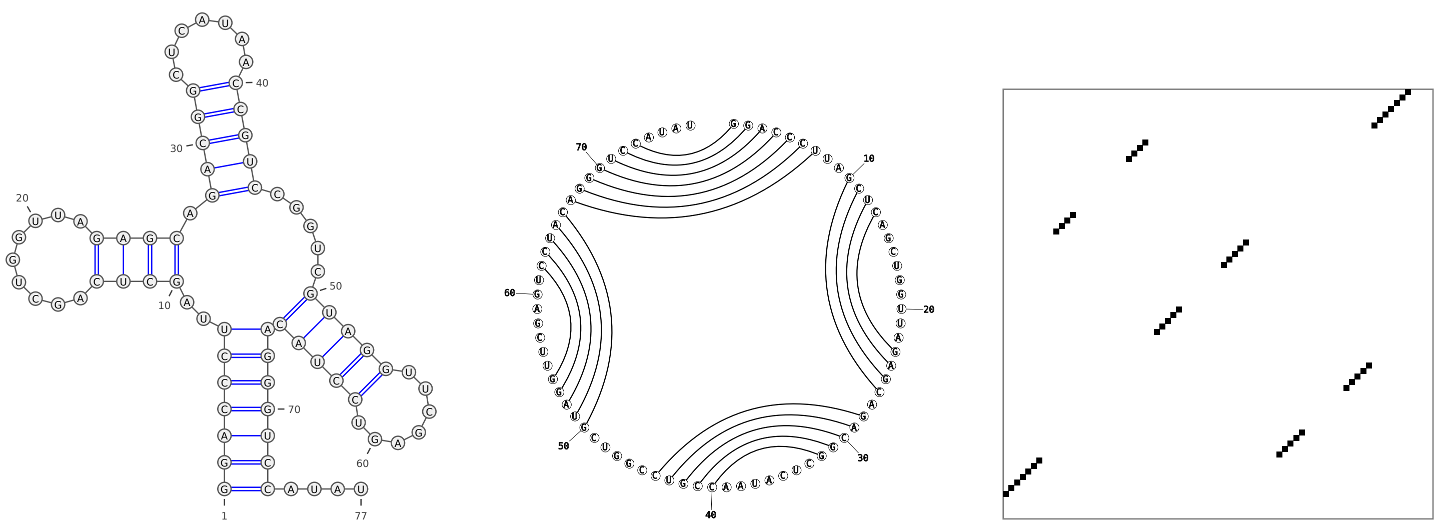

Lupinus luteus 3873 Ser CGA

Reference

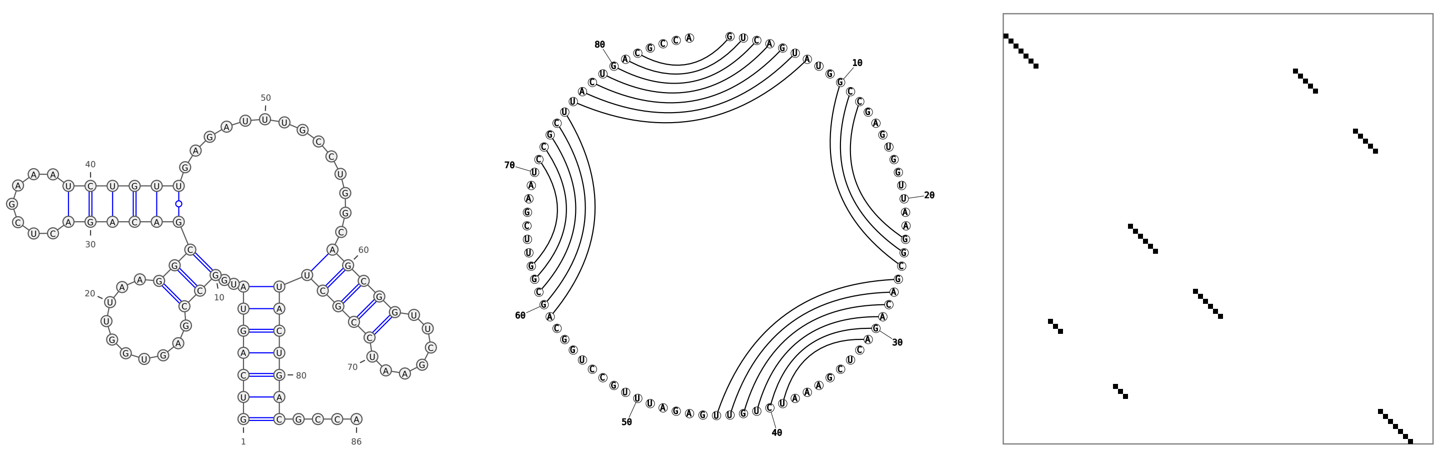

RNAfold $F_1 = 0.49$

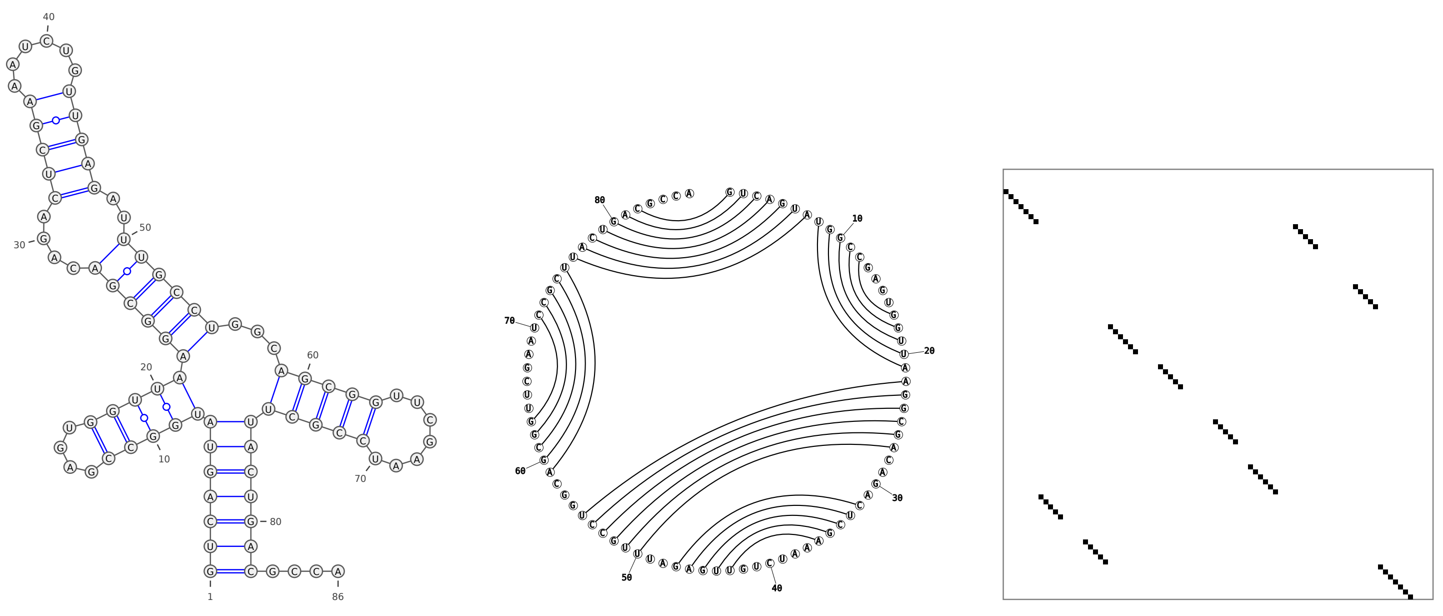

LinearPartition-C $F_1 = 0.77$

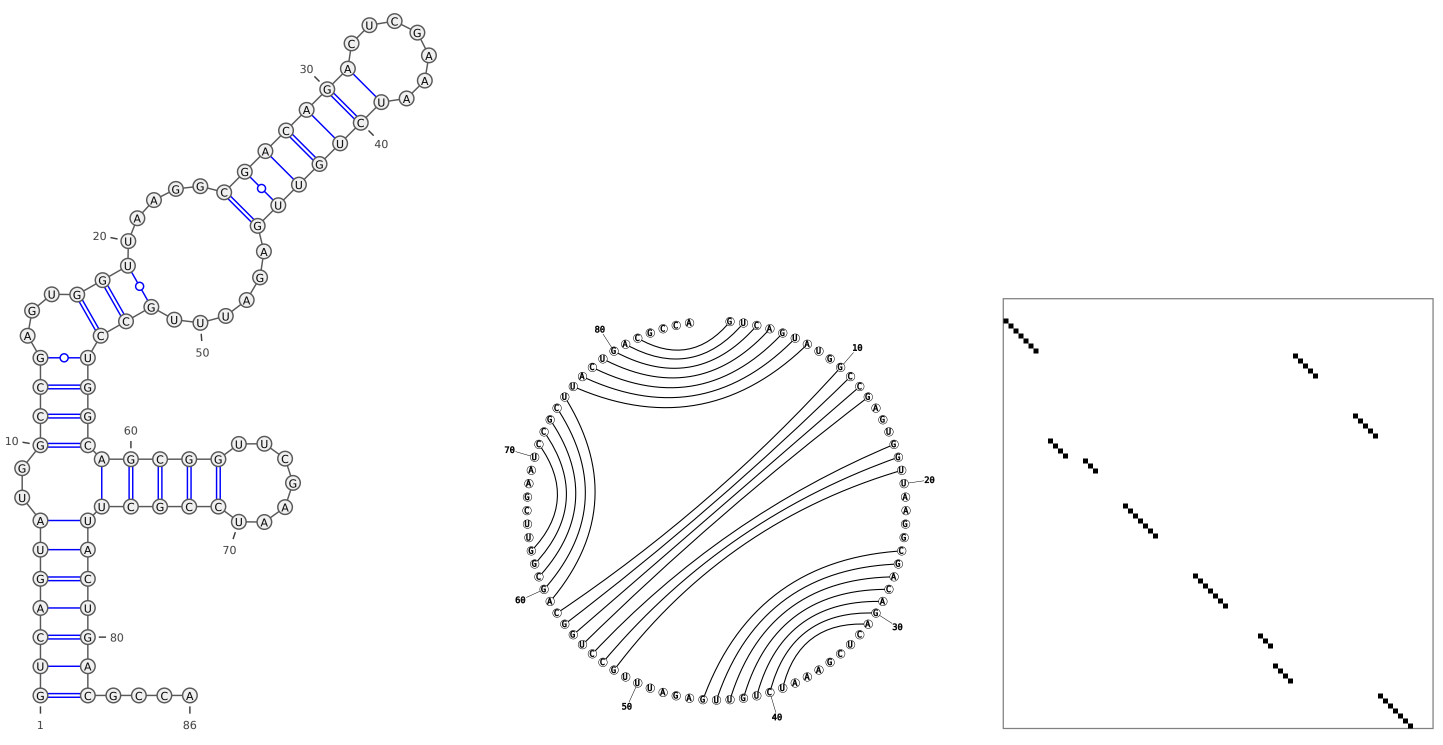

MXfold2 $F_1 = 0.75$

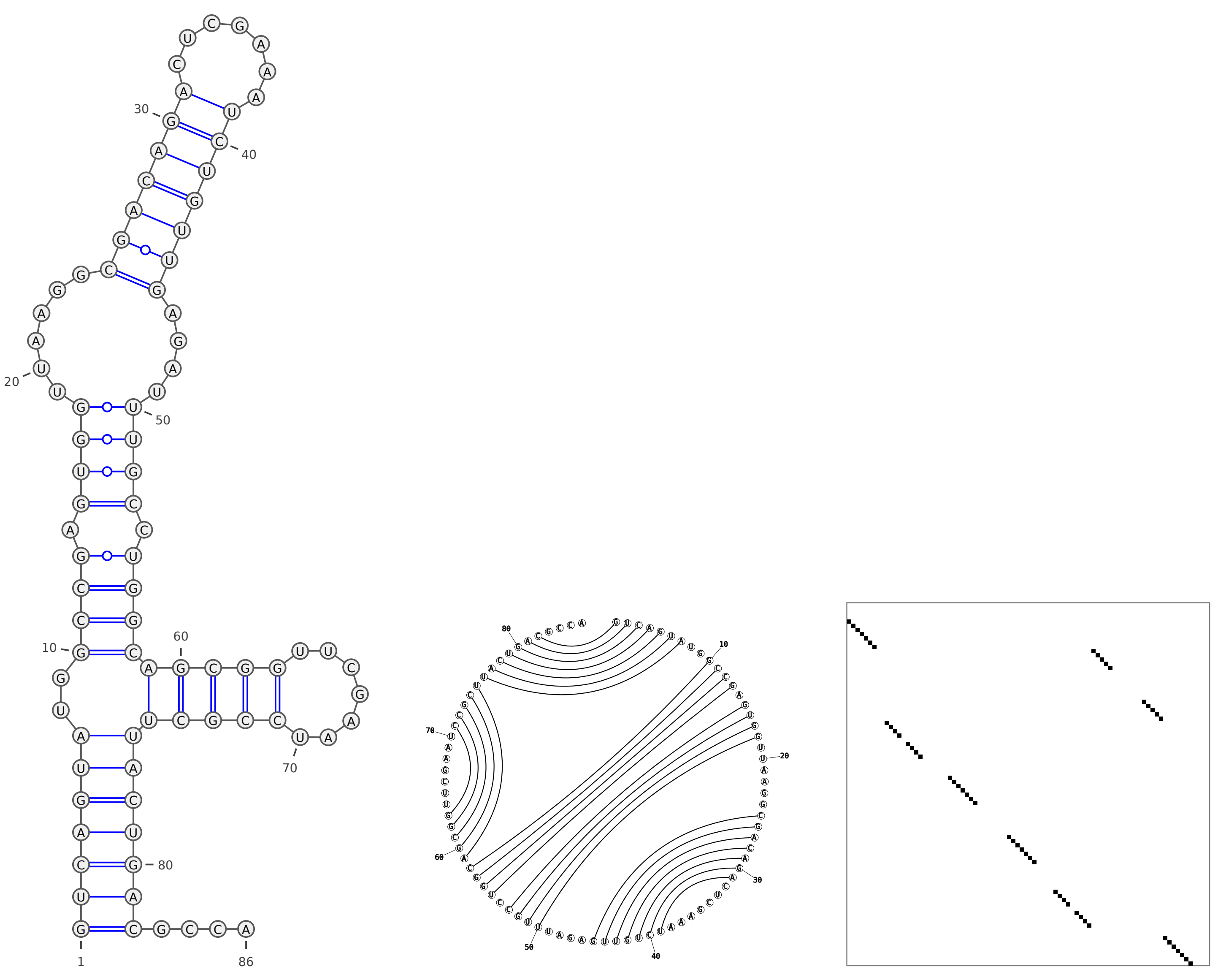

REDfold $F_1 = 0.73$

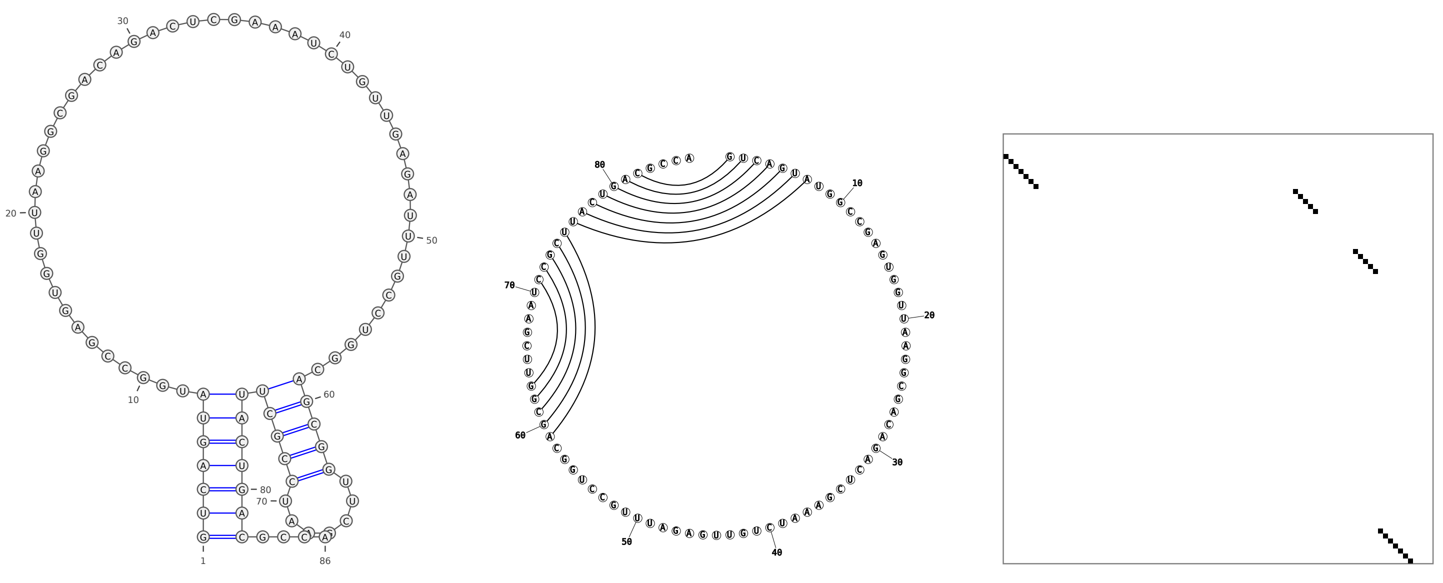

UFold $F_1 = 0.65$

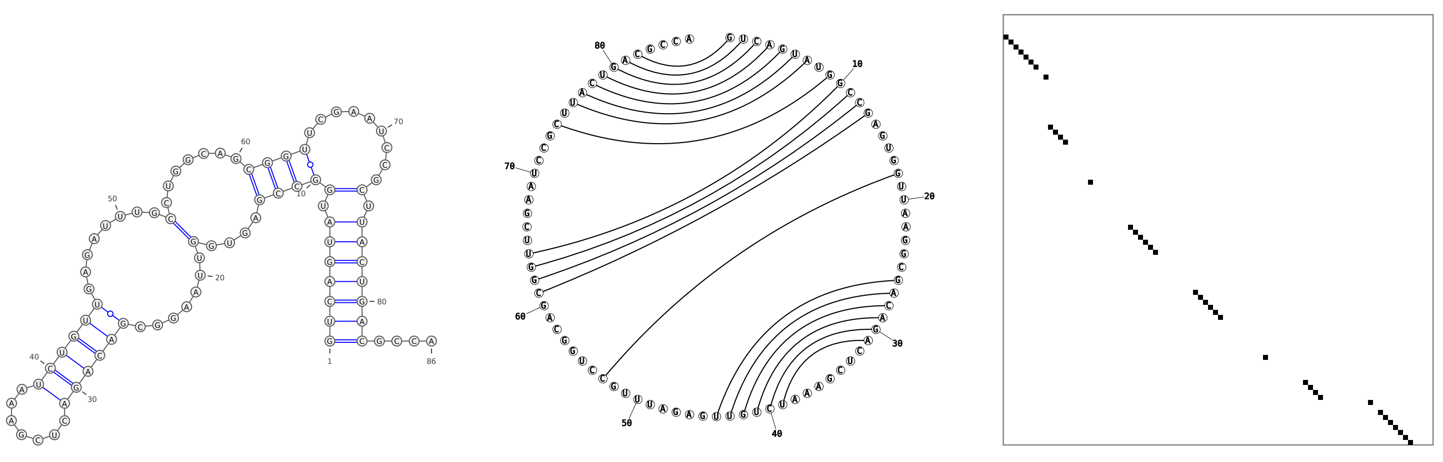

sincFold $F_1 = 0.90$

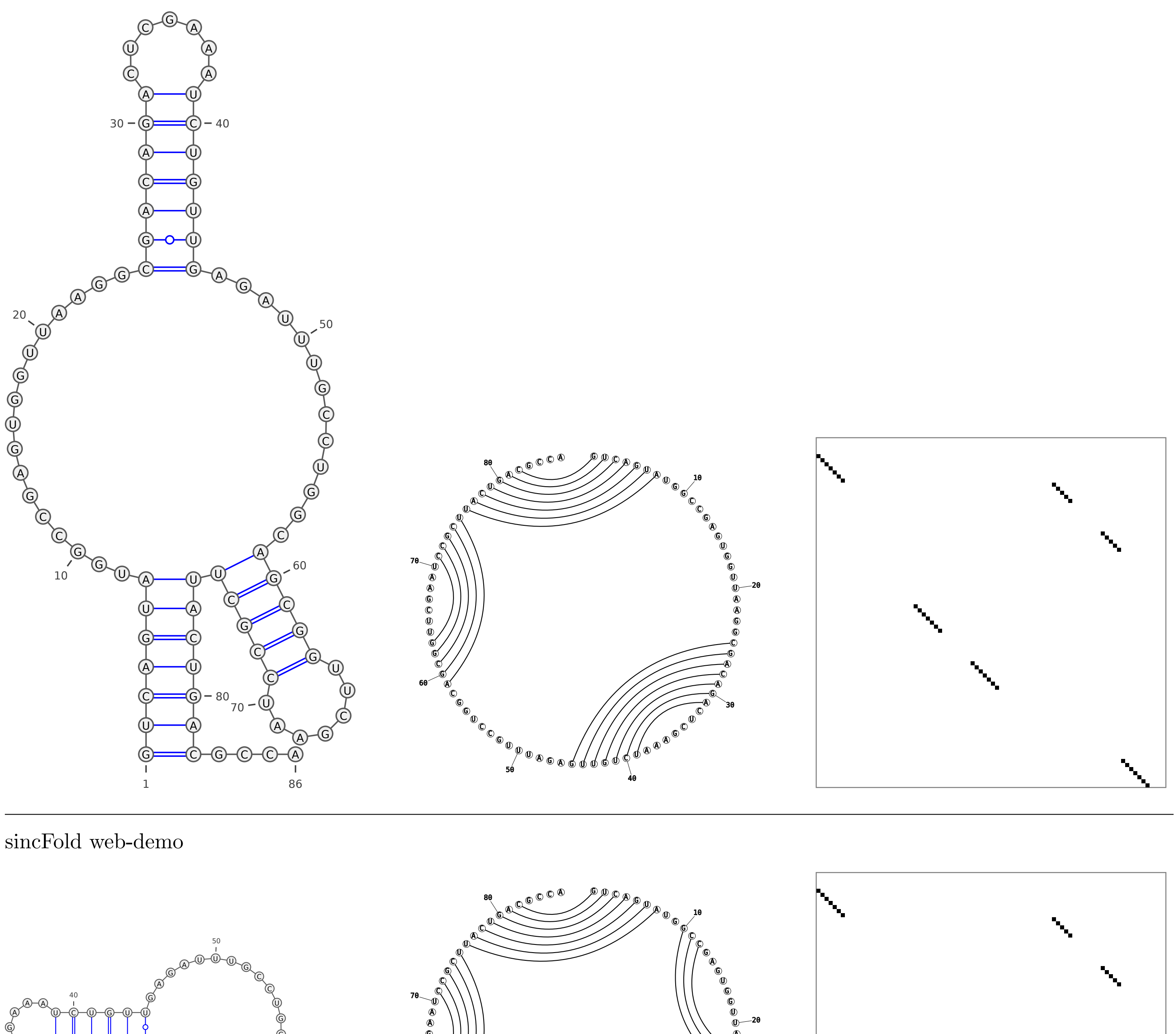

sincFold web-demo

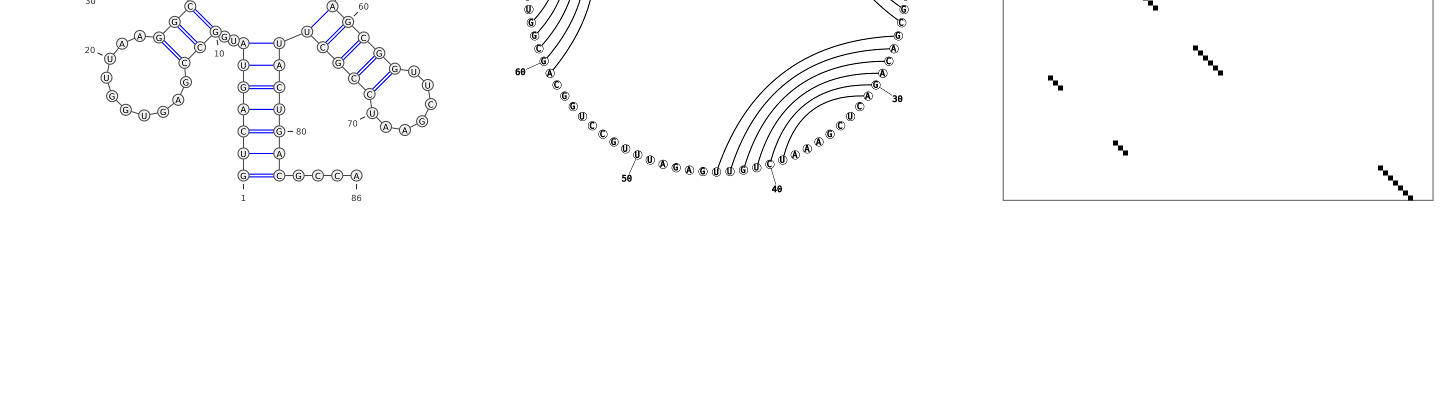

Drosophila melanogaster 7227 Ser GCU

Reference

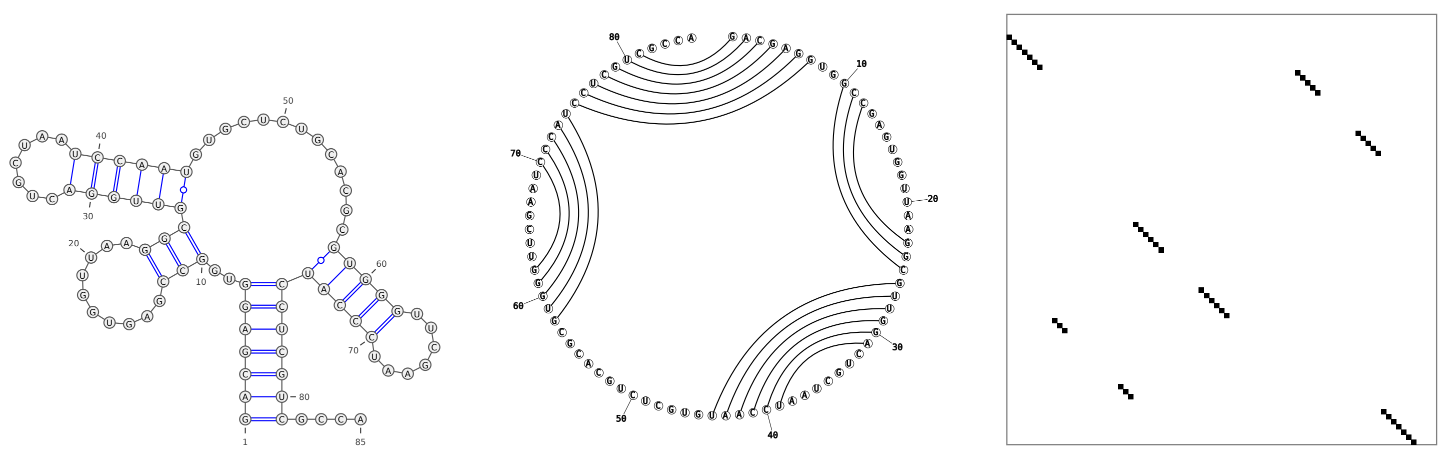

RNAfold $F_1 = 0.53$

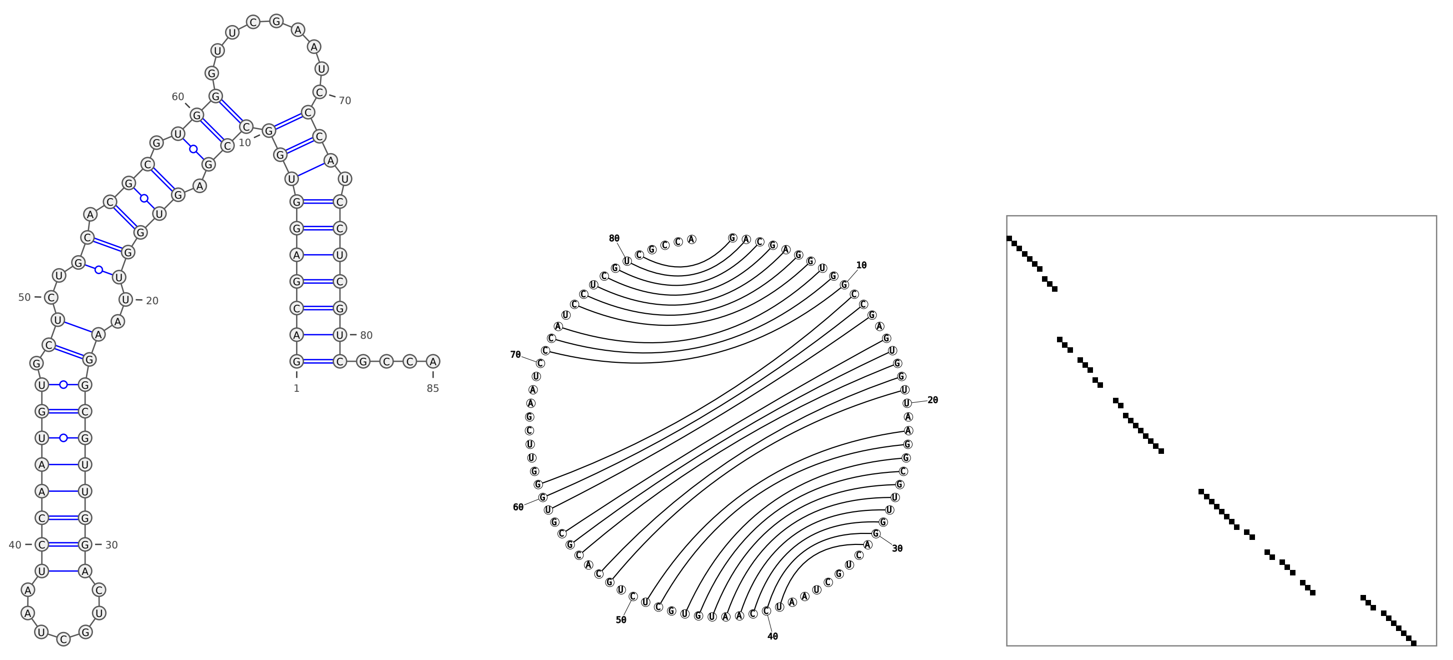

LinearPartition-C $F_1 = 0.73$

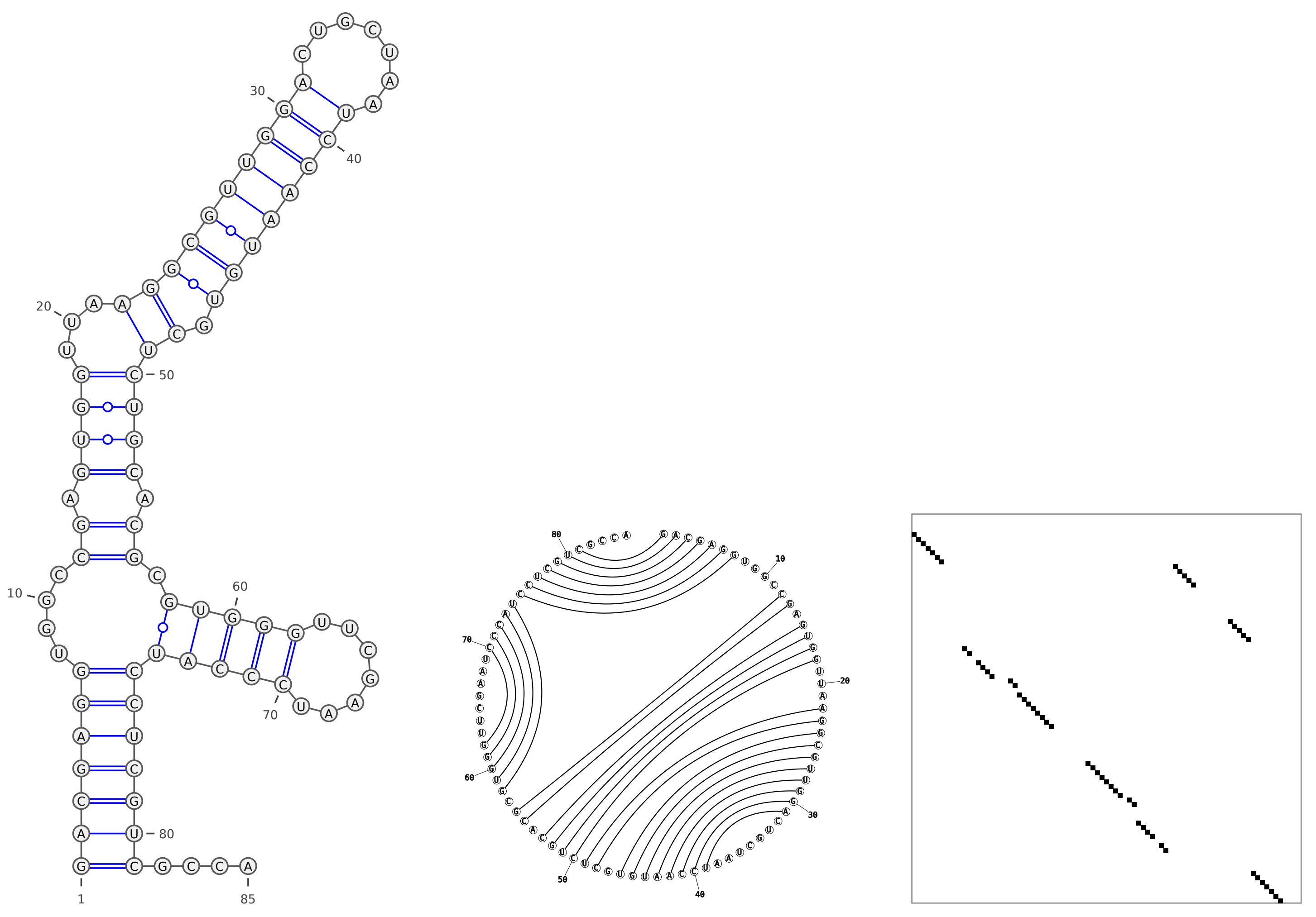

MXfold2 $F_1 = 0.73$

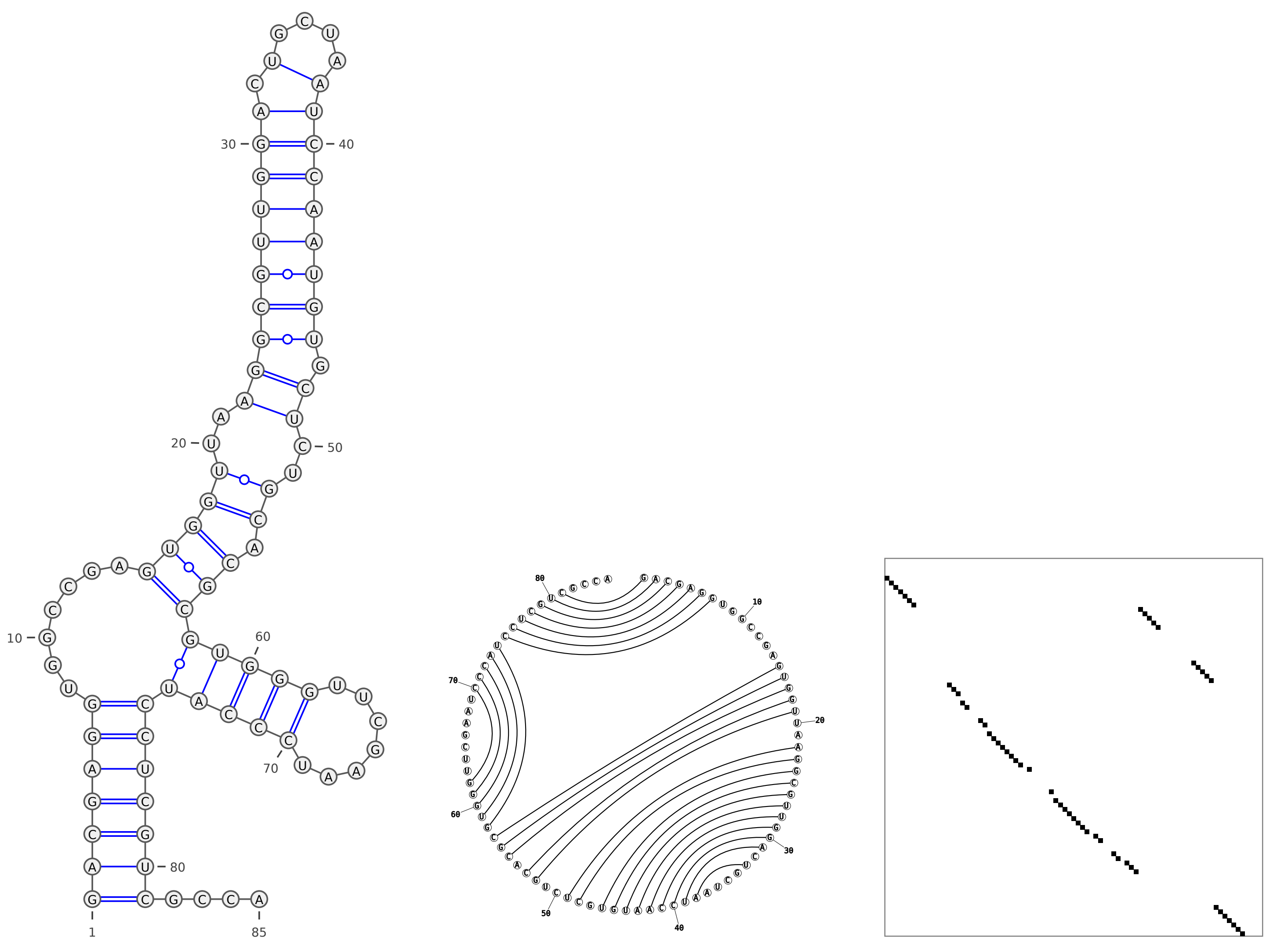

REDfold $F_1 = 0.60$

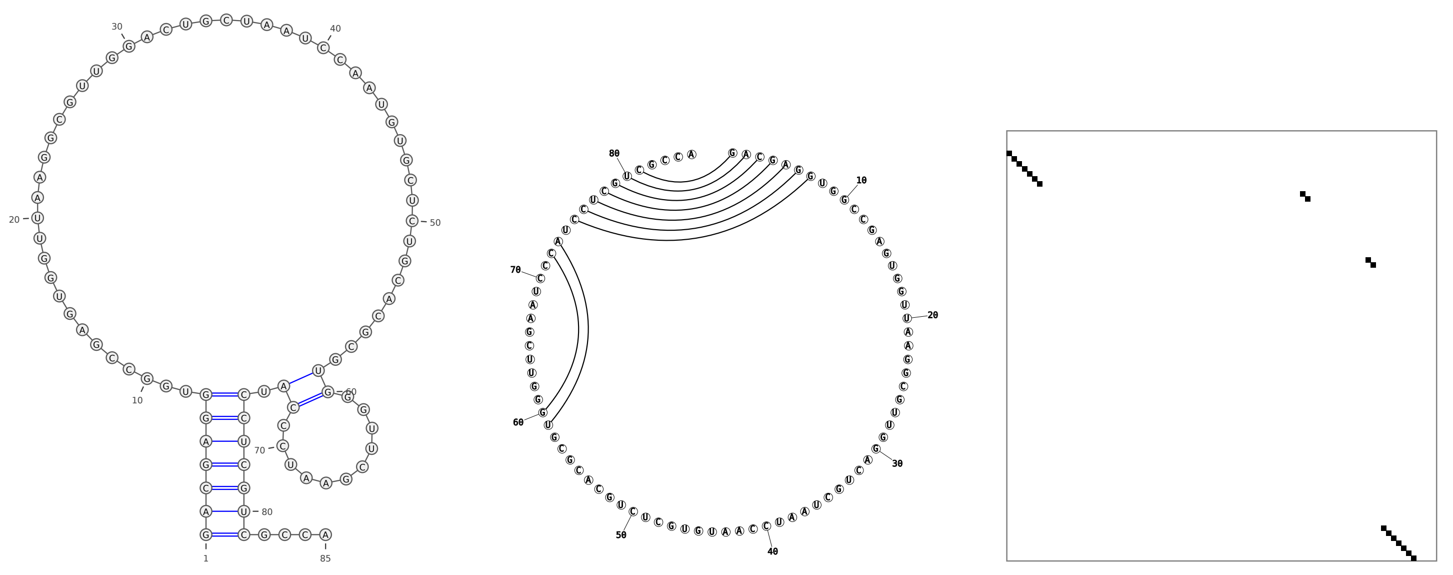

UFold $F_1 = 0.74$

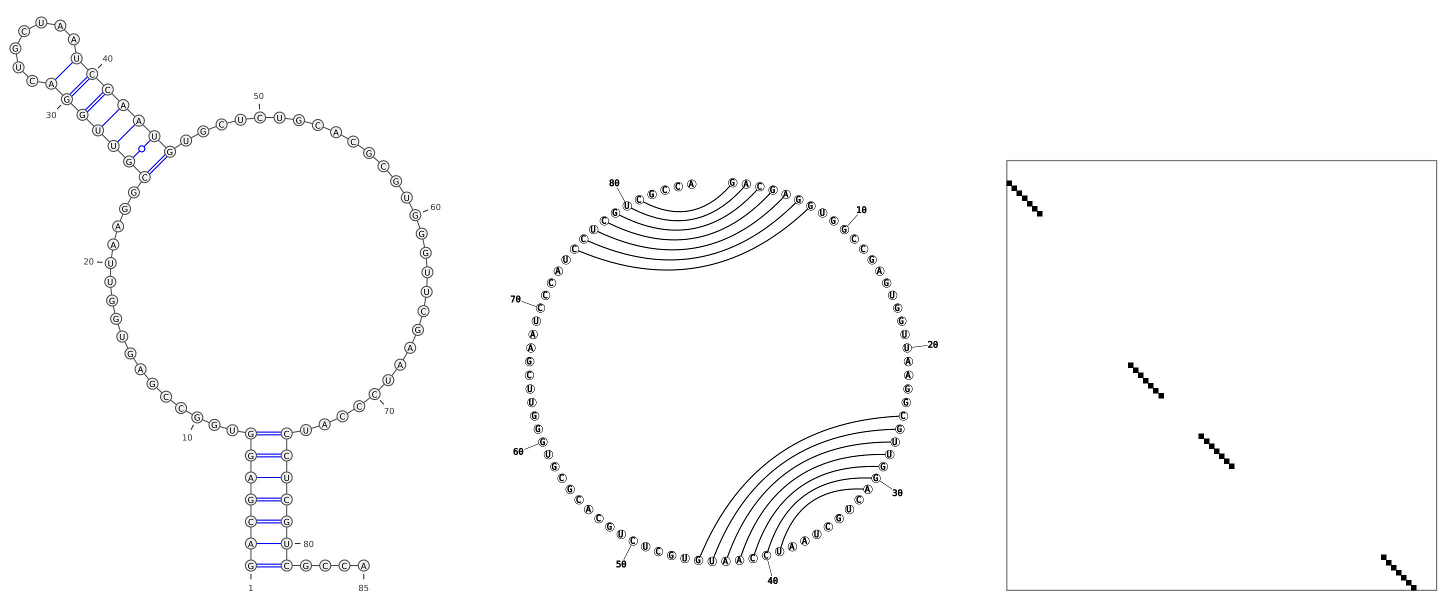

sincFold $F_1 = 0.80$

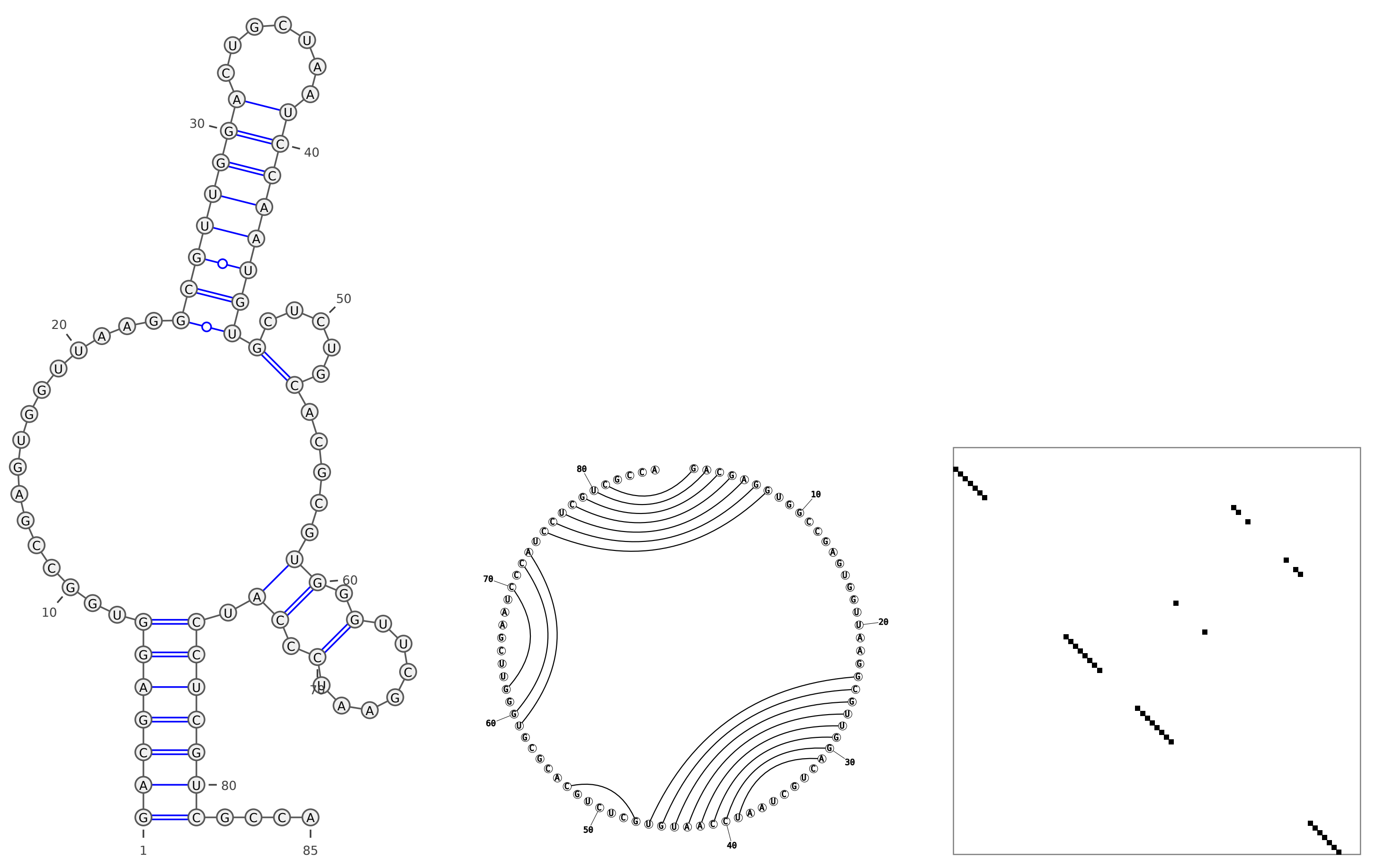

sincFold web-demo

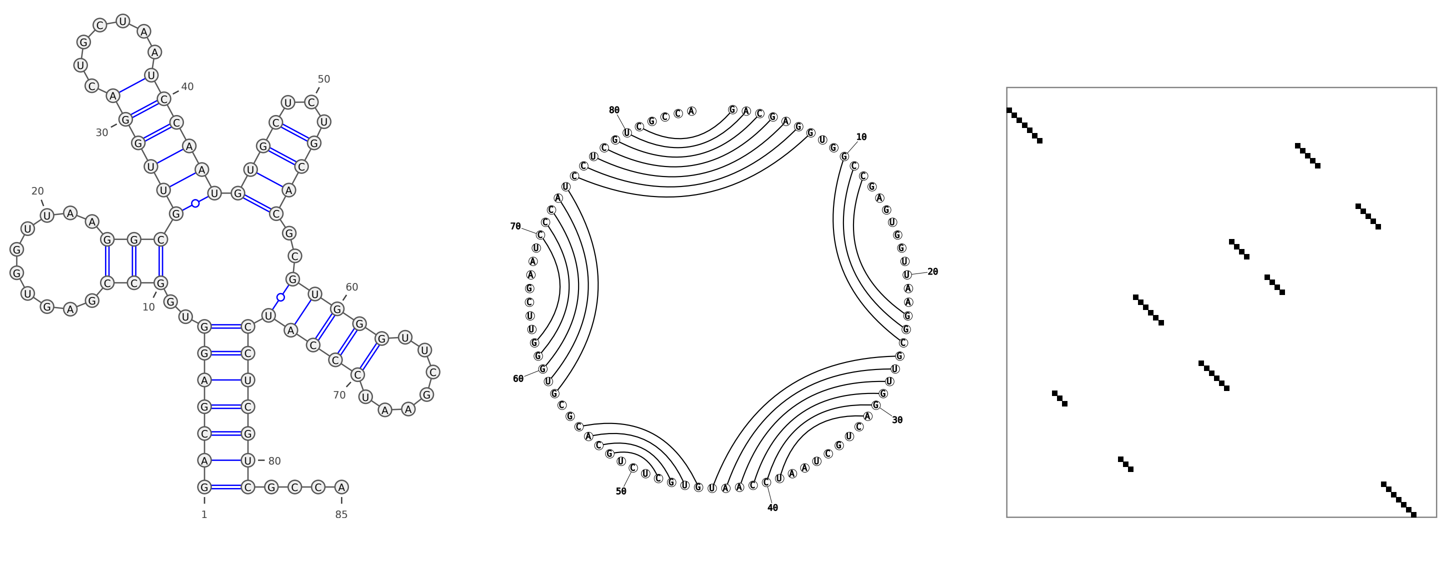

Supplement: sincFold_BiB_final_supmat_bbae271 [file sincfold_bib_final_supmat_bbae271.pdf]
